# Does conjugation strategy matter? Cetuximab-conjugated gold nanocages for targeting triple-negative breast cancer cells†

**DOI:** 10.1039/c9na00241c

**Published:** 2019-07-23

**Authors:** S. Avvakumova, L. Pandolfi, E. Soprano, L. Moretto, M. Bellini, E. Galbiati, M. A. Rizzuto, M. Colombo, R. Allevi, F. Corsi, A. Sánchez Iglesias, D. Prosperi

**Affiliations:** University of Milano-Bicocca, Department of Biotechnology and Bioscience Piazza della Scienza, 2 20126 Milano Italy davide.prosperi@unimib.it; Clinica di Malattie dell’Apparato Respiratorio, IRCCS Fondazione Policlinico San Matteo Pavia Italy; Dipartimento di Scienze Biomediche e Cliniche “Luigi Sacco”, Università di Milano via G.B. Grassi 74 20157 Milano Italy; Surgery Department, Breast Unit, ICS Maugeri S.p.A. SB via S. Maugeri 10 Pavia Italy; Nanomedicine Laboratory, ICS Maugeri S.p.A. SB via S. Maugeri 10 Pavia Italy; Bionanoplasmonics Laboratory, CICbiomaGUNE Paseo de Miramón 182 20014 Donostia-San Sebastián Spain

## Abstract

The efficient targeting of cancer cells depends on the success of obtaining the active targeting of overexpressed receptors. A very accurate design of nanoconjugates should be done *via* the selection of the conjugation strategy to achieve effective targeted nanoconjugates. Here, we present a detailed study of cetuximab-conjugated nonspherical gold nanocages for the active targeting of triple-negative breast cancer cells, including MDA-MB-231 and MDA-MB-468. A few different general strategies were selected for monoclonal antibody conjugation to the nanoparticle surface. By varying the bioconjugation conditions, including antibody orientation or the presence of a polymeric spacer or recombinant protein biolinker, we demonstrate the importance of a rational design of nanoconjugates. A quantitative study of gold content *via* ICP-AES allowed us to compare the effectiveness of cellular uptake as a function of the conjugation strategy and confirmed the active nature of nanoparticle internalization in cancer cells *via* epidermal growth factor receptor recognition, corroborating the importance of the rational design of nanomaterials for nanomedicine.

## Abstract 2

The efficient targeting of cancer cells depends on the success of obtaining the active targeting of overexpressed receptors.

## Graphical abstract

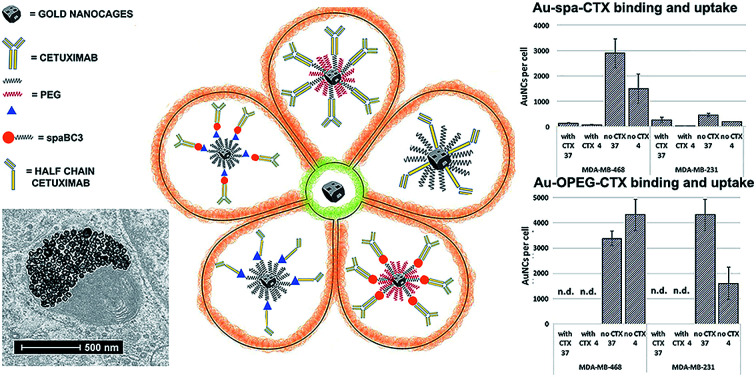

## Introduction

Tumor targeting is one of the main challenges in cancer nanotechnology.^1^ Numerous kinds of nanoparticles (NPs) with different sizes, shapes, and compositions have been developed; some of them have already been approved by the FDA for cancer treatment.^2,3^ However, a majority of treatment solutions involve the passive targeting of tumor tissues without implicating specific receptors. In contrast, active delivery using monoclonal antibodies (mAbs) as targeting ligands may improve the therapeutic effect of nanoconjugates by directly addressing NPs to cancer cells where the relevant receptors are overexpressed, while minimizing the effect on healthy tissues, thereby leading to reduced side-effects. Expectedly, this might improve the quality of life of patients.^4,5^ Nonetheless, in order to achieve efficient receptor targeting, a very accurate design of the nanoconjugate should be conducted. The choice of the conjugation strategy may play a fundamental role in the development of effective NPs, optimizing any preparation and characterization steps; an appropriate conjugation strategy should be used to obtain colloidally stable nanoconjugates and optimal targeting performance.^6^ This is particularly relevant when using advanced NPs, such as metal nanocages. This elegant class of colloidal NPs has demonstrated potential as innovative theranostic nanosystems useful for biomedical applications.^7^

In this project, we decided to investigate cetuximab (CTX)-conjugated cubic gold nanocages (AuNCs) as model NPs for epidermal growth factor receptor (EGFR) targeting in triple-negative breast cancer (TNBC) cells. Although the synthesis of such NPs is not straightforward since it requires multiple steps of synthesis and purification, we have already shown that AuNCs are a competitive tool for imaging because of their unique optical properties, which avoids the use of fluorescent dyes, and therefore, problems related to fluorescence bleaching and artifacts in biologic readouts related to the presence of organic dyes.^8,9^

Because of their strong absorption in the NIR region, AuNCs have become an alternative tool for imaging. CTX mAbs were used as the model molecule for conjugation to AuNCs, one of the most relevant mAbs in cancer therapeutics. CTX binds with high affinity and blocks the EGFR, which is overexpressed in several cancer cells.^10^ CTX-conjugated NPs have been widely used for the specific targeting of different kinds of cancer cells overexpressing EGFR, including lungs,^11–13^ gastric,^14^ colon,^15–17^ brain,^18–20^ and breast cancers.^21–23^

When a mAb is conjugated to a colloidal NP, the expected goal is to enhance the bioavailability, tumor-targeting efficiency, and ideally the therapeutic effect.^24^ A few critical issues should be taken into account while designing colloidal nanoconjugates for the targeting of molecular receptors, which include exercising control over the biomolecule/NP ratio, biomolecular orientation and activity, stability of the linkage between the NP and molecule, and, finally, the bioconjugation reaction conditions.^25–27^ The control over antibody orientation is probably the most difficult to achieve, yet one of the most relevant in order to optimize the targeting efficacy of nanoconjugates. Unfortunately, only a partial understanding of the impact of all these issues has been achieved until now.^25,28–31^ Several researchers have taken advantage of conventional conjugation approaches preferentially based on covalent immobilization, including amide coupling,^14^ thiol chemistry,^32^ and click reactions,^33^ as well as the direct adsorption of biomolecules onto NP surfaces regardless of their final orientation and protein folding.^34^ However, one conjugation strategy can effectively work for a certain mAb and a certain NP, while the same strategy can fail when using other mAbs and NPs. For this reason, developing controlled mAb bioconjugation onto NPs remains a challenge in nanobiotechnology.

A myriad of conjugation strategies have been formulated for NP functionalization. Probably, the most popular mAb conjugation method involves the coupling of a primary amine onto the antibody molecule, where EDC and sulfo-NHS are used as the coupling reagents. However, since the primary amine functional groups are randomly distributed throughout the antibody molecule, the arrangement of antibodies on a solid surface results in a random orientation, leading to diminished binding efficiency and selectivity associated with reduced accessibility of the antigen-binding sites on the mAb fraction.^35^ Moreover, the EDC/sulfo-NHS coupling system has often demonstrated poor conjugation efficiency: indeed, only 1–20% of the antibodies could be immobilized onto a solid surface by using this method.^36^

In the present study, we carefully designed mAb nanoconjugates following the various recently reported conjugation strategies, leading to either oriented or nonoriented CTX conjugation. The colloidal stability and antibody functionality of the NPs were verified throughout the synthesis process. Nanoconjugate uptake was quantified and compared as a function of conjugation strategy adopted in order to reveal the most suitable approach for CTX conjugation. Our approach has general utility: based on our results, we highlight the importance of undertaking preliminary research on the conjugation method to optimize NP biofunctionalization for the successful targeting of cancer cells.

## Results and discussion

The main idea of this project was to investigate the impact of the selection of the conjugation approach on the design and synthesis of mAb nanoconjugates. In particular, we aimed to show how the cellular uptake efficacy in different TNBC cells depended on the strategy used for CTX conjugation to colloidal NPs.

**Fig. 1:**
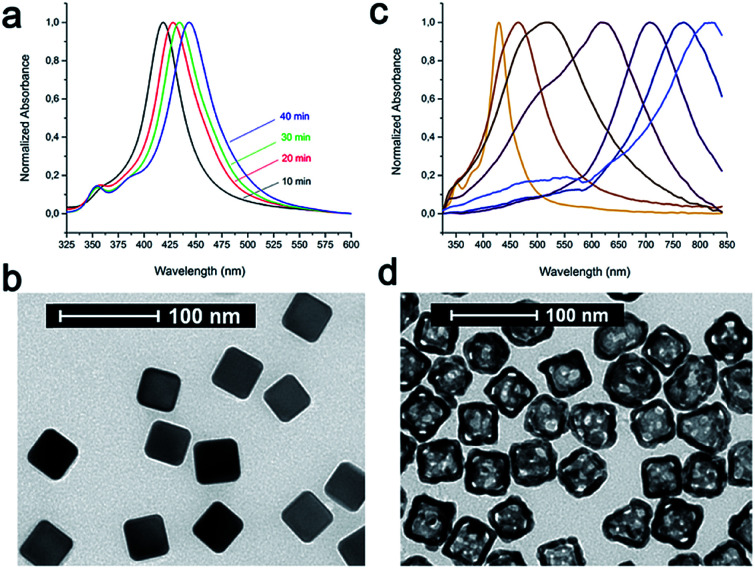
(a) UV-vis spectra of AgNC growth with SPR bands at 418, 428, 434, and 443 nm. (b) Transmission electron microscopy (TEM) image of AgNCs (side length: 40.4 ± 2.8 nm). (c) UV-vis spectra of the starting AgNCs and AuNCs during galvanic replacement reaction. (d) TEM image of AuNCs with side length of 44.5 ± 3.0 nm and gold shell of 7.0 ± 1.2 nm.

As model NPs, AuNCs (44.5 ± 3.0 nm) were synthesized by the galvanic replacement reaction starting from silver nanocubes (AgNCs; side length: 40.4 ± 2.8 nm) (Fig. 1). AgNCs were synthesized using CF_3_COOAg as the precursor in accordance to the polyol synthesis process, as described by Zhang *et al.*^37^ These AgNCs were found to grow in size at a controllable pace over the course of the synthesis, and their sizes in terms of the edge length were controlled by the position of the SPR band, while their quality was checked by the determination of the full width at half maximum (FWHM): lower values indicated better batch quality. The localized surface plasmon resonance (LSPR) band of each batch was tuned to 435–445 nm; however, it was challenging to obtain the batches with the same values of LSPR maxima, while the reaction time could vary from 20 to 50 min depending on the batch. Importantly, all the solutions were used as freshly prepared for better reproducibility of the nanocubes. At the end of the synthesis, all the samples were collected by centrifugation and then washed once with acetone to remove the remaining precursors and ethylene glycol (EG), followed by washing three times with ethanol to remove excess polyvinylpyrrolidone (PVP). Finally, these AgNCs were kept in ethanol and used as soon as possible. We highlight that different batches of AuNCs have been used for these studies, due to a huge variety normally encountered in preparing these samples (NP size and LSPR band position could slightly shift from one preparation to another), and all the experiments reported in this work were repeated several times to confirm the batch-to-batch reproducibility. The NP concentration was measured by nanoparticle tracking analysis (NTA) and expressed in NPs per mL.

The main advantage of using AuNCs rather than spherical gold NPs is because of their optical properties, which allowed us to exploit their intrinsic transmittance emissions for cellular uptake studies by confocal microscopy, thereby avoiding contamination from dye labeling.^9^

In addition, AuNCs could be exploited for the conversion of irradiated energy into heat upon light absorption in the NIR region, thereby behaving like nanomediators in photothermal therapy.^8^ CTX was chosen as the biorecognition molecule because of its prevalent use for therapeutic and diagnostic targeting processes for ErbB1 receptors in several tumor malignancies, including breast cancer.^38^

**Scheme 1:**
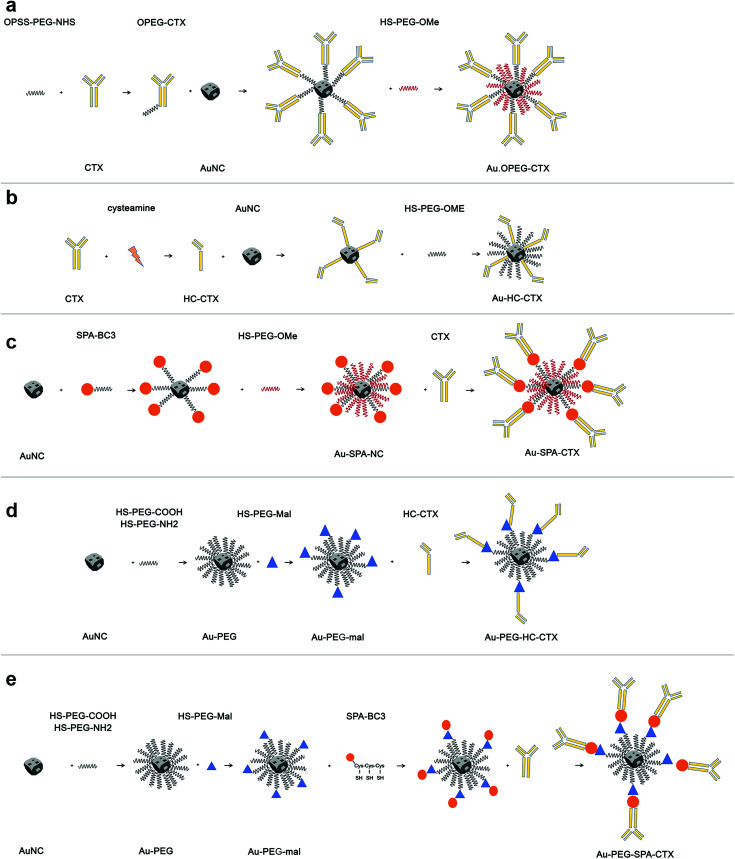
Conjugation strategies involving AuNCs and CTX reported in this paper. (a) CTX immobilization by using a PEG-NHS spacer; (b) direct immobilization of HC-CTX exploiting the thiol groups derived from the rupture of disulphide bridges between the mAb heavy chains; (c) CTX immobilization by using a spa-BC3 biolinker; (d) HC-CTX conjugation using a PEG-mal spacer; (e) CTX immobilization by using spa-BC3 with a PEG-mal spacer.

The first part of this study was dedicated to the optimization of the composition of AuNCs ligand shell and development of an appropriate synthesis protocol to obtain stable nanoconjugates. Among the several bioconjugation strategies available from the literature, we have selected a few to functionalize AuNCs with CTX, which facilitated a broader generality (see Scheme 1).

The first strategy of choice involved the semi-oriented conjugation of mAbs to NPs through a polyethylene glycol (PEG) spacer. In this method, the primary amine groups on CTX were chemically modified with a 2 kDa orthopyridyldisulfide polyethylene glycol *N*-hydroxysuccinimide ester (OPSS-PEG-NHS ester) by reaction with the NHS group. Next, the PEGylated CTX was conjugated to AuNCs *via* the Au–S bond of the *ortho*-pyridyl disulphide (OPSS) group, yielding Au-OPEG-CTX NCs (Scheme 1a). The functionalized AuNCs were further coated with a saturating amount of HS-PEG-OMe to improve the nanoconjugate colloidal stability.

Different researchers have shown that NP stability and targeting efficiency directly depend on the ratio between the targeting molecule and PEG coating on the NP surface.^39,40^ Within this strategy, we could choose between two different protocols for CTX conjugation. In the first protocol, AuNCs could be sequentially reacted with OPSS-PEG-mAb, followed by HS-PEG addition,^41^ while the second one involved the simultaneous addition of OPSS-PEG-mAb and shorter HS-PEG to AuNCs.^42^ On the basis of the research of Bergeron *et al.*, where these two synthetic variants were compared, we preferred to use the sequential strategy for CTX conjugation, which resulted in more stable NPs with the highest receptor recognition efficacy of the antibody.^43^ CTX was first functionalized with PEG at the ratio of 1 : 20 (CTX : PEG). Assuming that an IgG molecule contains 83 lysine residues on average,^44^ PEG functionalization was aimed to cover only a fraction of them, without compromising the antigen recognition ability of CTX.^44^ The typical dimensions of IgG were approximately 14.5 nm × 8.5 nm × 4.0 nm,^44^ where 14.5 nm is the size of the largest portion between the antigen-binding sites.

On the basis of these parameters, the projected area of the IgG molecule on the NP surface should be 3.14 × (7.25 nm)^2^ = 165 nm^2^. The total surface area of a cube with a side of 45 nm is 6 × (45 nm)^2^ = 12 150 nm^2^; therefore, 1 NP can accommodate around 73.6 CTX molecules. For this reason, we used a marginally excess amount of CTX in the conjugation reaction, considering a ratio of 1 : 100 (NP : CTX).

As shown by Puntes *et al.*, when the conjugation of CTX is performed in a random manner without considering the ratio between the antibody and NP, multilayers of antibodies can be formed, ultimately entrapping several NPs and compromising both colloidal stability of nanoconjugates and mAb functionality.^28^ Finally, taking into account the fact that the grafting density of one PEG_2000_ molecule is 1.07 ± 0.02 PEG per nm^2^,^45^ as well as considering the abovementioned total surface area of the nanocube, we needed at least 11 355 PEG molecules to fully cover the NP surface. In order to afford a complete coating, we used large PEG excess (100 000 PEG molecules per NP) corresponding to 8.8 thiolated PEG chains for each site available on the AuNC surface. These assumptions and calculations were also considered in the other conjugation strategies investigated in this study.

In the second strategy, a half-chain CTX (HC-CTX) was conjugated to AuNCs with higher control over the molecular orientation. HC-CTX fragments were obtained by the reduction of disulfide bridges that hold the two mAb chains.^46^ Then, the reduced thiol groups were used in the reaction with AuNCs, as shown in Scheme 1b. This method, previously tested in our laboratory using different NPs,^47,48^ showed promising results, allowing for the immobilization of mAb fragments with a fixed orientation that could preserve the intact affinity toward the specific molecular receptor (EGFR, in this case). Indeed, disulfide bridges that bind the two mAb halves are located at the ends of the constant fraction (Fc), in an area sufficiently far away from the fragment involved in the receptor recognition (Fab). The reduction of the disulfide bonds was effected by reacting the antibody with the 2-mercaptoethylamine (cysteamine) reducing agent, as shown in Scheme 1b. CTX-HC produced in this way was immediately reacted with AuNCs in order to prevent the oxidative reassociation of the antibody. As that in the earlier method, Au-HC-CTX NCs were finally saturated with HS-PEG_2000_-OCH_3_.

The third strategy took advantage of the affinity between a specially engineered protein (spa-BC3)^49^ and the constant portion of human IgG for the conjugation of monoclonal antibodies to gold NPs.^50^ This method has already been used by our group for the immobilization of trastuzumab antibody (Tz, Herceptin®) on the surface of spherical NPs, yielding encouraging results.^47,49,50^ Spa-BC3, as shown in Scheme 1c, is an engineered variant of the B domain of protein A,^51^ which has the ability to specifically bind to the constant portion (Fc) of human IgG with high affinity.^52^ To allow its interaction with AuNPs, a tripod of cysteines was conveniently inserted at the C-terminal end, along with a histidine tag (Hisx6) useful for protein purification.^50^ The advantage of using spa-BC3 for the conjugation of CTX to AuNCs relies on the fact that the antibody can be immobilized on the surface with a specific and suitable orientation of Fab, since the binding between spa-BC3 and CTX engages the antibody Fc fragment.

The selectivity of spa-BC3 binding to therapeutic mAbs (*e.g.*, Tz) has already been shown in our earlier research.^50^ As shown in Scheme 1, spa-BC3 was subsequently reacted with AuNCs, exploiting the high affinity of cysteine tripod toward gold, followed by the reaction with CTX and PEG saturation using HS-PEG_2000_-OCH_3_. To study the effect of molecular mobility on the NP, we investigated the recognition between CTX and EGFR by increasing the distance of CTX from the AuNC surface using a suitable short spacer to separate the antibody. AuNCs were first coated with a mixture of HS-PEG_2000_-COOH and HS-PEG_3000_-NH_2_ at a ratio of 75 : 25%. These experimental conditions were found to induce the maximum stability toward the NPs, given that the amino groups could undermine AuNC colloidal stability.

Next, an NHS-PEG_8_-maleimide heterobifunctional linker was added to Au–NH_2_–COOH NPs in order to further conjugate HC-CTX and spA-BC3 proteins by reaction with lysine residues. These two additional strategies are shown in Scheme 1d and e, respectively.

An additional strategy adopted for mAb nanoconjugation comprised the oxidation of a saccharide residue (carbohydrate-containing *cis*-diol groups) present in the Fc portion of the antibody.^5,53^ This procedure comprised the formation of an aldehyde group capable of reacting with nucleophilic functionalities (such as amines) that are appropriately visible on the NP surfaces, as shown in Fig. S1.† This method also allowed us to bind CTX to AuNCs along a specific orientation through its constant portion, without compromising the Fab region and ensuring the maintenance of its functionality.^28^ Antibody oxidation and the functionalization of NPs with amine groups were conducted in separate steps: first, the antibody was treated with the oxidizing agent NaIO_4_; in the second step, the NPs were reacted with a mixture of two bifunctional PEGs, namely, HS-PEG_2000_-OCH_3_ and HS-PEG_3000_-NH_2_, both exhibiting the thiol functionality that can to bind to the NP surface. The reaction between the aldehyde group formed on the oxidized antibody (SugOX-CTX) and the amine groups on the surface of the functionalized NPs formed a Schiff base, which was subsequently reduced by the addition of NaCNBH_3_ (Fig. S1†). The reactivity of SugOX-CTX toward the secondary antibody was also checked by the dot blot analysis, confirming its maintained activity after oxidation (Fig. S1†).

The CTX-conjugated nanocages were characterized by UV-vis spectroscopy and dynamic light scattering (DLS) analysis, determining their hydrodynamic diameter and zeta potential. According to the literature,^54^ the conjugation with CTX caused a red-shift of 12–17 nm in the LSPR band for all the samples (Table S1†), due to a variation in the NP extinction coefficients. These shifts were independent of the initial LSPR peak position. The UV-vis spectra of Au-OPEG-CTX, Au-CTX-HC, and Au-spa-CTX are shown in Fig. S2.† With regard to Au-SugOX-CTX (absorption spectrum shown in Fig. S3†), the conjugation with HS-PEG_3000_-NH_2_ (Au-PEG-NH_2_) caused a net widening of the LSPR band, indicating a certain loss of colloidal stability. This instability was recovered even after conjugation with CTX. On the contrary, all the other conjugates maintained their stability, demonstrating a peak of width similar to that observed in nonconjugated NPs.^55^ On the basis of the stability data, we did not investigate the SugOX-CTX conjugation strategy any further, focusing the biological investigation on the methods that yielded more stable nanoconjugates. DLS and zeta potential studies yielded good results, reporting different variations in the hydrodynamic sizes after protein conjugation and marginal zeta potential increase (from −26.0 ± 2.7 mV (PVP-coated AuNCs) to less negative values) (Table S2†). Worthwhile polydispersity indexes of all the nanoconjugates confirmed their stability during the analyses.

Further colloidal stability studies in different media showed worthwhile stability profiles for all the nanoconjugates under physiological conditions, in serum, and at different pHs (Fig. S4†), as evident from the UV-vis spectra and their shapes before and after incubation with the media. The nanoconjugates were incubated with the medium for 1 h prior to the UV-vis measurements. The worthwhile stability of the nanoconjugates remains an obvious characteristic that should be confirmed for their application in nanomedicine.

**Fig. 2:**
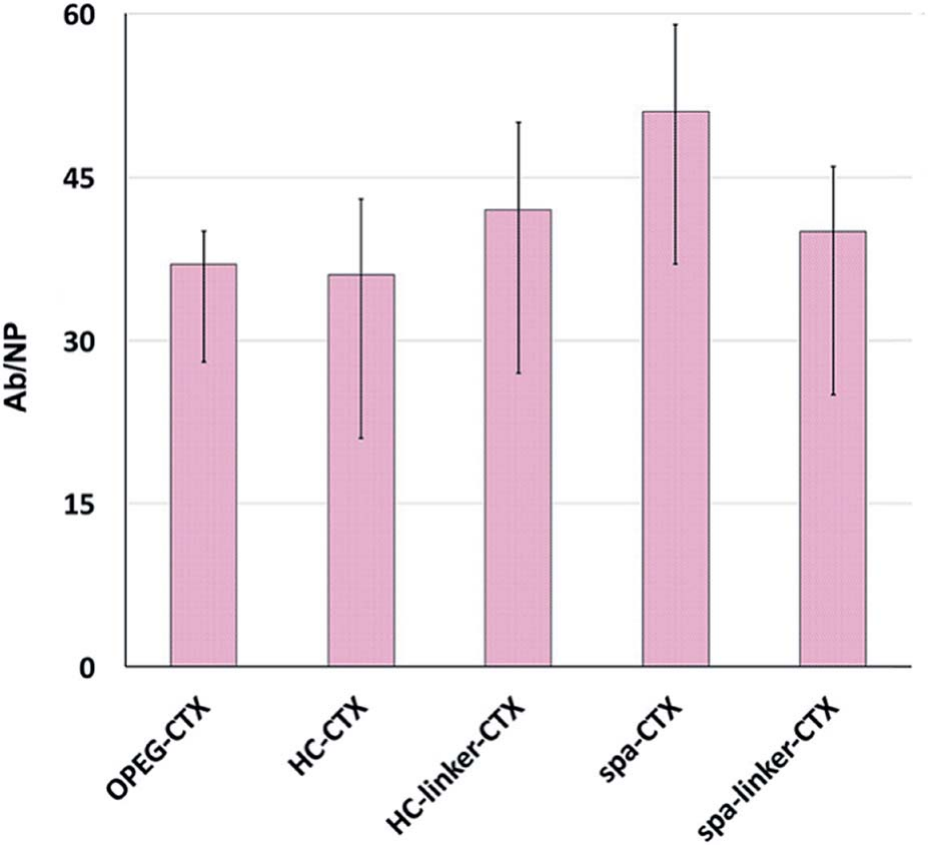
Number of CTX per AuNC measured by the indirect Bradford analysis: 37 Abs for Au-OPEG-CTX, 36 Abs for Au-HC-CTX, 42 Abs for Au-HC-linker-CTX, 51 Abs for Au-spa-CTX, and 40 Abs for Au-spa-linker-CTX. The data were presented as mean ± s.d. (*n* = 6).

Protein loading was analyzed after CTX conjugation. The amount of conjugated CTX on each AuNC was indirectly calculated by subtracting the amount of free CTX determined by the Bradford analysis from the CTX concentration of the initial solution. The number of CTX molecules attached to the surface of AuNCs in each nanoconjugate type is shown in Fig. 2. Unfortunately, since the analysis was performed on at least six different batches of NPs, there was significant variability. This can be due to the slightly different sizes of the as-synthesized NP batches, which, in turn, resulted in different total surface areas available to the host CTX. Nevertheless, in the medium, all the conjugation strategies yielded very similar results in terms of the number of CTX molecules per particle, ranging between 36 and 51 mAbs per NP.

**Fig. 3:**
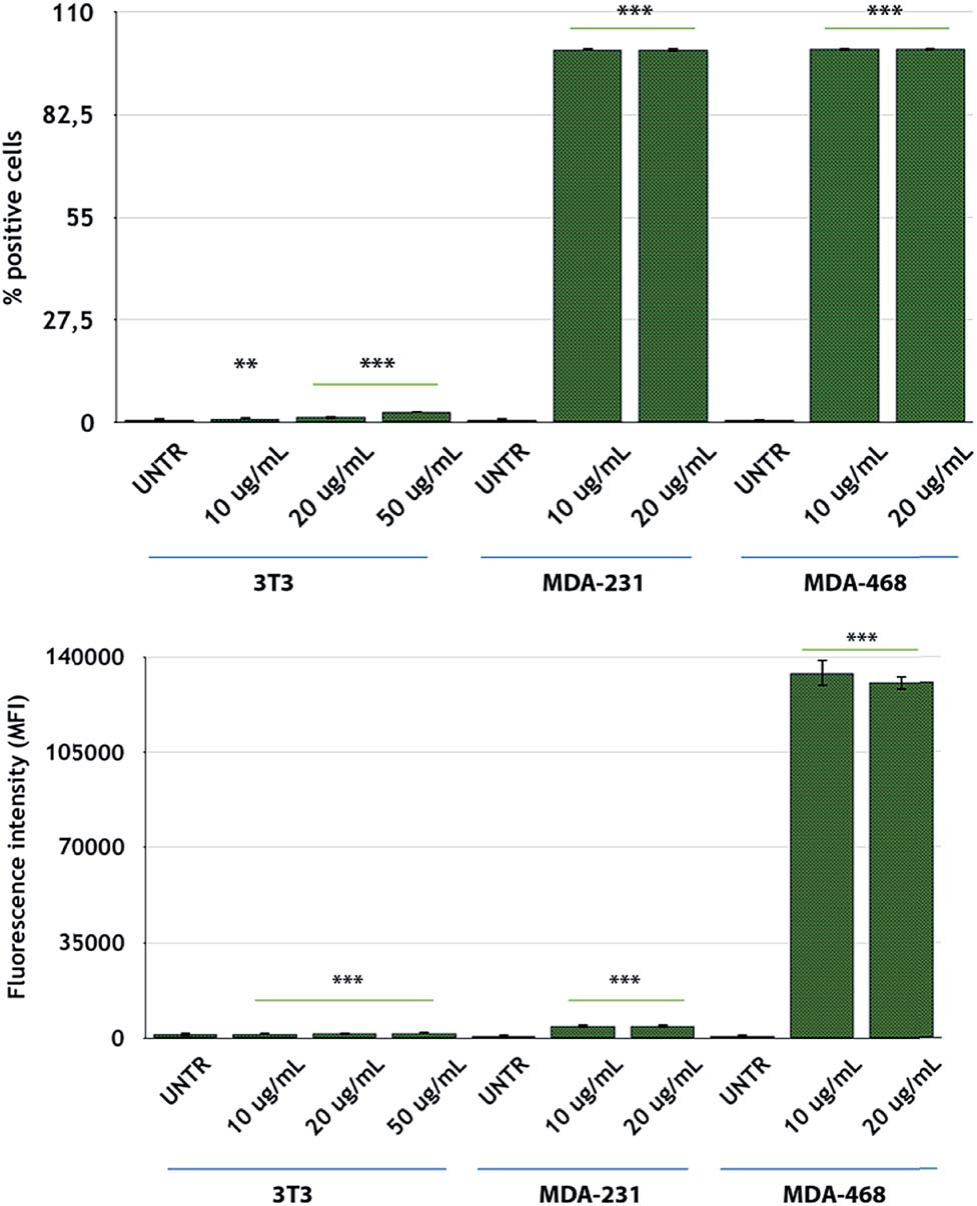
FACS analysis of CTX-FITC binding and EGFR expression levels in MDA-MB-231, MDA-MB-468, and 3T3-L1 cells. Reported values are mean ± s.d. (*n* = 3). *p < 0.05; **p < 0.005; ***p < 0.001 *vs.* UNTR (Student's *t* test).

After NP characterization, the diverse nanoconjugate systems were evaluated by *in vitro* experiments using TNBC cells. Two breast cancer cell lines overexpressing the EGFR receptor, namely, MDA-MB-231 and MDA-MB-468 cells,^56^ were selected for this study, while 3T3-L1 murine fibroblast cells were used as the healthy control cell line. To confirm the EGFR expression in these TNBC cells, we conducted flow cytometry analysis of the cells incubated with fluorescein-labeled CTX to saturate the EGFR receptors. MDA-MB-468 cells showed the highest levels of EGFR expression, according to the highest fluorescence levels, as compared to that for MDA-MB-231 cells (Fig. 3). On the other hand, 3T3-L1 control cells showed the lowest EGFR expression level. These data are in accordance with the data reported by Jeong *et al.*^57^

Finally, we compared the cellular uptake of each nanoconjugate in TNBC cells by inductively coupled plasma atomic emission spectroscopy (ICP-AES) analysis with the aim to investigate the bioconjugation strategy that yields the highest AuNC internalization. MDA-MB-231 and MDA-MB-468 cells were incubated for 24 h with 0.4 and 0.8 nM AuNCs by using five different conjugates. After incubation, the cells were carefully washed to eliminate any unbound NPs and digested in aqua regia.

**Fig. 4:**
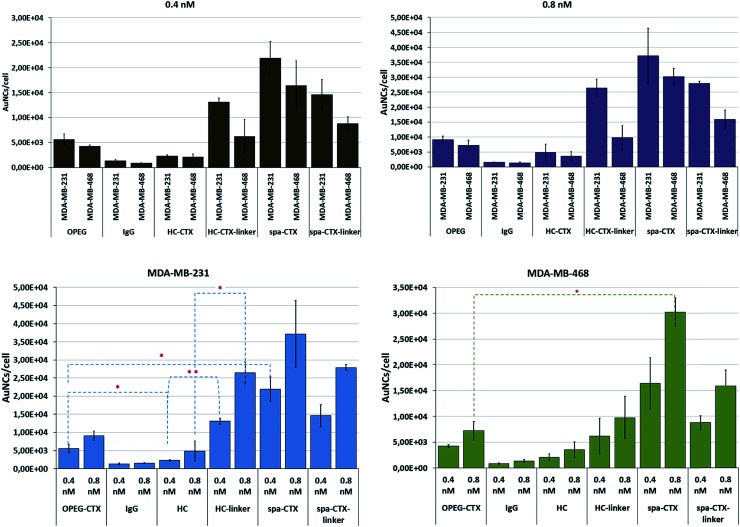
Cellular uptake of AuNCs conjugated with CTX by using different conjugation approaches. Gold content per cell was analyzed by ICP-AES, and the number of NPs per cell was calculated. The data are represented as different combinations for better understanding of the results. Reported values are mean ± s.d. (*n* = 3). *p < 0.05; **p < 0.01; ***p < 0.005 *vs.* UNTR (Student's *t* test).

The number of particles per cell was calculated based on the amount of gold found by ICP and correlated with the NP concentration found by NTA. By comparing this data with the results from the analysis of NP batches at known concentrations, we obtained the Au content per NP; by proportion, the total number of NPs per cell was determined. The results are reported as means of at least 6 individual experiments with relative standard deviation (Fig. 4). Although MDA-MB-231 cells exhibited lower EGFR density as compared to MDA-MB-468 cells (10^5^*vs.* 10^6^ receptors per cell),^33^ the uptake was slightly higher in MDA-MB-231 cells for each nanoconjugate at both these concentrations. However, these results referred to the amount of AuNCs incorporated by cells at 24 h and do not necessarily reflect the uptake kinetics. From this viewpoint, the apparent higher uptake in MDA-MB-231 cells could be explained in terms of the different kinetics of internalization, sorting, and excretion of NPs. The uptake kinetics was influenced by not only the ligand density and NP size, but also the receptor density itself. When the concentration changed from 0.4 to 0.8 nM, the cellular uptake proportionally increased, indicating a concentration-dependent mechanism of NP internalization in both the cell lines. Au-IgG NCs, used as the control for unspecific cellular uptake, showed the lowest number of NPs per cell in both the cell lines, and this amount was independent of the cell type.

With regard to the cellular uptake efficiency as a function of the conjugation method, we could conclude that at 24 h of incubation, Au-spa-CTX NCs showed the highest uptake, with a total of about 21 900 and 37 100 AuNCs per cell for MDA-MB-231 and 16 400 and 30 200 AuNCs per cell for MDA-MB-468 at NP concentrations of 0.4 and 0.8 nM, respectively. The insertion of a linker between the protein and NP surface did not seem to improve the cellular uptake, yielding lower values of gold content. However, a thorough statistical analysis of the results did not show any significant difference in the uptake before and after linker insertion. This can be due to the high variability of data as a result of the experiments with different NP batches. Moreover, a high number of steps during the synthesis can induce variability. On the other hand, excessive PEGylation can lead to a strong inhibition of cellular uptake and a less efficient interaction and binding with the target receptors; this is because the target recognition of ligand molecules located on the surfaces of PEGylated NPs is also dependent on the density and thickness of the PEG layer and the positioning of the ligand.^58^

The lowest uptake was obtained when using Au-HC-CTX, with around 2290 (at 0.4 nM) and 4830 (at 0.8 nM) AuNCs per cell in MDA-MB-231 cells and 2090 (at 0.4 nM) and 3570 (at 0.8 nM) AuNCs per cell in MDA-MB-468 cells. Even if this uptake seemed very low as compared to those in the other samples, the overall amount of NPs engulfed by cells was high when compared with earlier published data.^57^ It is noteworthy that the conjugation of HC-CTX to the NP surface *via* a linker significantly improved the total uptake at 24 h in both the cell lines. In contrast, this confirmed our hypothesis that the direct conjugation of an antibody to such charged NPs could damage its conformation and compromise the correct interaction with the cell receptor. However, as long as the antibody was distanced from the NP surface by a short PEG linker, the charge interference decreased, allowing for a more properly adapted interaction with the EGFR receptor. Eventually, Au-OPEG-CTX NCs, where CTX was conjugated in a nonspecific manner, exhibited intermediate cellular uptake as compared to the other conjugates investigated in this study. However, the preparation of this nanoconjugate was more straightforward as compared to the other ones, requiring less effort and lower number of synthesis steps. Altogether, the above considerations suggested that the sample reproducibility and biological results could be directly influenced by preparation issues. In fact, when comparing the standard deviations of the uptake data, Au-OPEG-CTX (and Au-IgG, prepared in the same way) afforded the lowest values. On the basis of this, the ideal nanoconjugate for biological investigations should afford the best compromise among three fundamental issues, *i.e.*, cellular uptake, reproducible results, and straightforward synthesis. Finally, considering PEGylated NPs, it is supposed that the PEG extends away from the particle. However, there are two regimens for polymers attached to the surface, depending on their grafting density. If the density is too low, the polymer is said to be in a “mushroom-like” conformation, where the PEG chains bend toward the surface. However, if the grafting density is sufficiently high, the polymers are said to be in the “brush-like” regimen, and the PEG molecules extend away from the surface. The molecular weight of the polymer, as well as its grafting density, determines the degree of surface coverage and distance between the graft sites, and they ultimately affect the exposure of the targeting moiety and NP-targeting efficiency.^59^

The results for the EGFR^−^ 3T3-L1 nontumor cells are shown in Fig. S5.† The uptake of nanoconjugates was not concentration-dependent and showed similar values regardless of the conjugation strategy adopted. Only one nanoconjugate (Au-spa-CTX-linker) exhibited enhanced interaction with cells, probably because of the higher level of nonspecific uptake. On the basis of the above results, we decided to limit the subsequent steps in this study only to Au-OPEG-CTX and Au-spa-CTX nanoconjugates.

**Fig. 5:**
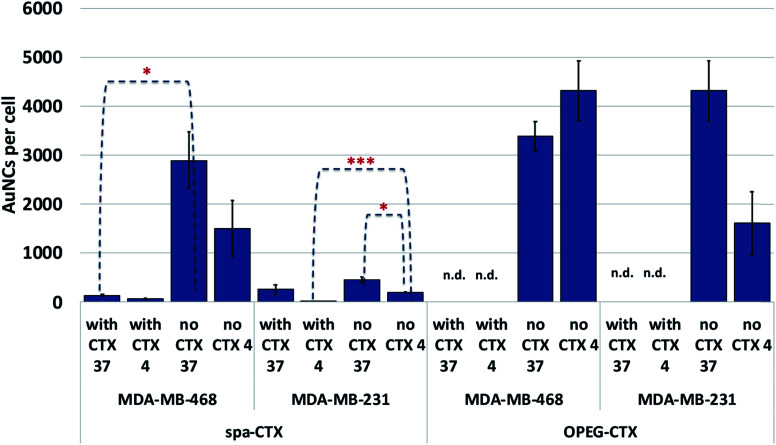
Cellular binding and uptake of Au-OPEG-CTX and Au-spa-CTX nanoconjugates at 4 and 37 °C in the presence and absence of free CTX. Reported values are mean ± s.d. (*n* = 3). *p < 0.05; **p < 0.01; ***p < 0.005 *vs.* UNTR (Student's *t* test).

We determined the binding selectivity of Au-OPEG-CTX and Au-spa-CTX NCs toward EGFR in the three cell lines by means of ICP-AES. To this aim, the cells were separately incubated with 0.8 nM nanoconjugates for 1 h at 4 °C, thoroughly washed, and the amount of gold was analyzed to determine the number of AuNCs per particle. The results are shown in Fig. 5. In order to confirm that Au-OPEG-CTX and Au-spa-CTX NCs were internalized by the cells specifically *via* the EGFR receptor, we performed competition studies with CTX. The cells were preincubated with unlabeled CTX (1 mg mL^−1^) for 30 min at 4 °C and then incubated with CTX-conjugated AuNCs (0.8 nM, 1 h) at 4 °C. As shown in Fig. 5, after blocking the EGFR receptor with CTX at 4 °C, which rigidified the cellular membrane, the number of targeted NPs inside the cells drastically dropped. This implied that the interactions between the NPs and MDA-MB-468 or MDA-MB-231 cells were inhibited when the EGFR receptors were competitively bound by excess CTX molecules. When the receptors were free to bind with the nanoconjugates (at 4 °C), Au-spa-CTX NCs showed higher binding in MDA-MB-468 cells as compared to that in MDA-MB-231 cells, which is in accordance with the relative extent of EGFR expression determined by flow cytometry (Fig. 3). The same conclusion could be drawn using Au-OPEG-CTX NCs in the absence of free CTX at 4 °C. When comparing the two nanoconjugates after 1 h of incubation, Au-OPEG-CTX apparently yielded the highest binding ability in both the cell lines, probably because of faster binding kinetics. In some cases, we could hardly detect the amount of gold in the samples as it was under the detection limit of the instrument (n.d.: not determined). These data can be considered equal to zero, confirming the specificity of the binding between the nanoconjugates and corresponding receptors.

When the incubation was conducted at 37 °C, we observed a marginal increase in NP content inside both the EGFR-overexpressing cell lines due to the more rapid uptake kinetics at this temperature. The only exception was Au-OPEG-CTX NPs incubated with MDA-MB-468 cells, which showed a higher uptake at 4 °C rather than that at 37 °C. Moreover, these conflicting results were not supported by statistics, suggesting that the levels of cellular uptake could be very similar. We have two hypotheses in this regard: a high level of NPs were nonspecifically uptaken, or the kinetics or cellular uptake at 37 °C is so slow in this cell line that it is comparable to the binding kinetics at 4 °C. When the receptors were saturated by CTX, the cellular uptake decreased, indicating the receptor-mediated characteristic of the internalization as the preferred uptake mechanism. In EGFR-negative 3T3 cells, both NP binding at 4 °C and uptake at 37 °C after 1 h of incubation were very low or even negligible, and they were hardly influenced by the CTX pretreatment of cells, confirming the nonspecific characteristic of the interaction (Fig. S6†).

**Fig. 6:**
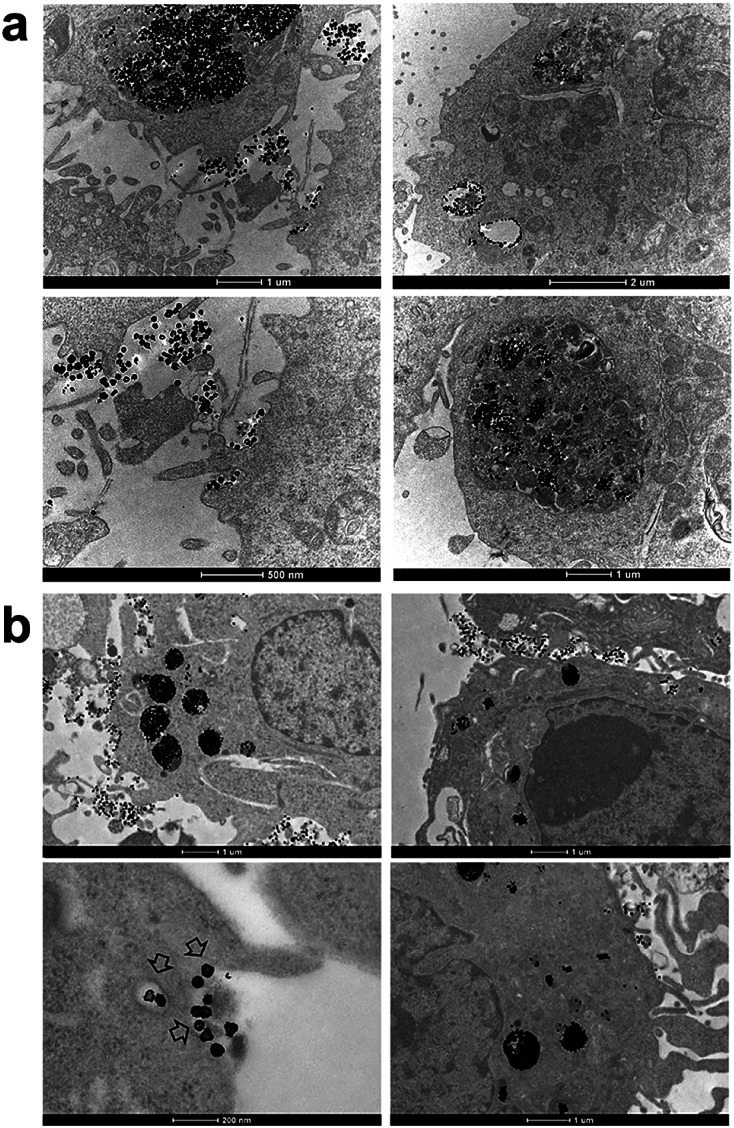
TEM images of Au-OPEG-CTX NCs incubated for 24 h in (a) MDA-MB-231 and (b) MDA-MB-468 cells.

**Fig. 7:**
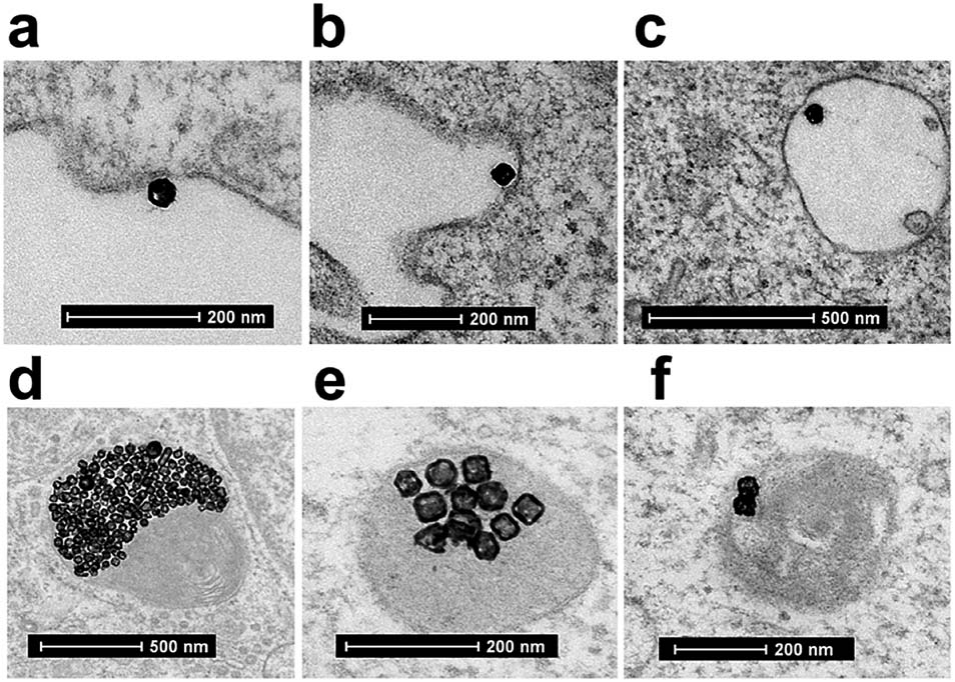
TEM image of cellular uptake mechanism of Au-OPEG-CTX NCs by MDA-MB-468 cells. (a) First step is a clathrin-mediated interaction of nanoparticles with cell membrane, (b) which triggers the formation of an early endosome, (c) followed by internalization of the nanoparticle into the mature endosome. (d) Next step is the accumulation of clusters of nanoparticles in endosomes, (e and f) progressively evolving to lysosomes.

Finally, we performed the TEM analyses for the cellular uptakes of Au-OPEG-CTX and Au-spa-CTX NCs in TNBC cells in order to assess the particle interactions with the cell membranes and trafficking inside the cells. The cells were incubated for 24 h and processed to obtain slices for TEM visualization. As evident from Fig. 6, Au-OPEG-CTX NPs were distributed either in the extracellular matrix or inside the endosomes and lysosomes in huge amounts. Interestingly, the vesicles seemed to be bigger in size in MDA-MB-231 cells as compared to those in MDA-MB-468 cells. The size of endosomes including the NPs could influence their performance in photothermal applications *in vitro*, with larger vesicles yielding stronger plasmon coupling effects, and therefore, higher temperature rise.^60^ In Fig. 7, we propose an example of internalization mechanism for Au-OPEG-CTX NCs.

**Fig. 8:**
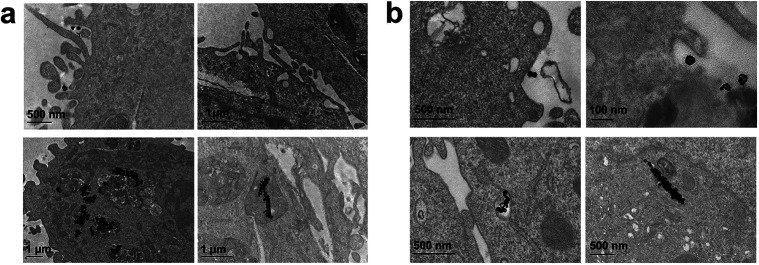
TEM images of Au-spa-CTX NCs incubated for 24 h in (a) MDA-MB-231 and (b) MDA-MB-468 cells.

A clathrin-mediated interaction of NPs with a cell membrane was hypothesized, followed by endosome formation leading to the accumulation of a huge number of NPs in the lysosomes. This confirmed the receptor-mediated endocytosis mechanism of internalization, in accordance with the results of the competition (binding) studies, as shown in Fig. 5. Au-spa-CTX generally showed a similar internalization mechanism, where smaller vesicles were formed during NP uptake (Fig. 8). It is noteworthy that the receptor-mediated mechanism of uptake was often accompanied by other internalization mechanisms, such as micropinocytosis, particularly when excess NPs were used in the experiments; this made it difficult to rule out parallel uptake routes to enter the cell.

## Conclusions

In summary, we have reported the results of a wide and detailed study on how the selection of conjugation strategy involving broadly utilized targeting biomolecules, such as mAbs, can affect the uptake rates of AuNCs in TNBC cells. To show the importance of the rational design of nanoconjugates for *in vitro* studies, we synthesized AuNCs containing anti-EGFR CTX by using five different bioconjugation strategies. By quantitatively comparing the NP uptake in MDA-MB-231 and MDA-MB-468 TNBC cells as well as that in 3T3-L1 healthy cells measured by using ICP-AES, we concluded that the nanoconjugate preparation procedure significantly affected the experimental success in cell cultures, where the number of intermediate conjugation steps or the possibility of using a spacer could indirectly influence the interaction with the target cells of the final nanoconjugate. Among the five different nanoconjugates selected for this study, two of them, namely, Au-spa-CTX and Au-OPEG-CTX, yielded a good compromise between the stability and uptake rates in cells. Based on this result, the cellular uptake mechanism was studied. Competition and binding studies in cells at 4 and 37 °C revealed the occurrence of energy-dependent receptor-mediated endocytosis in EGFR-overexpressing cells. Although this study is of fundamental interest for scientists looking for an effective way to conjugate mAbs to AuNCs, it is important to consider that the expected biological results *in vitro* or *in vivo* depend on the NP preparation philosophy, revealing the importance of conjugation strategy in nanomedicine. Future works should be devoted toward exploring the generality of our conclusions using NPs of different sizes and shapes.

## Experimental

### Synthesis of AuNCs

AuNCs were prepared by a galvanic replacement reaction between AgNCs and HAuCl_4_ in an aqueous solution, following the protocol reported by Zhang *et al.*^37^ and Skrabalak *et al.* that was slightly modified.^61^ In a standard synthesis process of AgNCs, ethylene glycol (10 mL, EG) was added into a 100 mL round-bottom flask and heated under magnetic stirring in an oil bath preset to 158 °C. Freshly prepared NaSH (0.160 mL, 3 mM in EG) was quickly injected into the heated solution after its temperature reached 158 °C. Thereafter, a certain volume of HCl solution (1 mL, 3 mM in EG) was injected into the heated reaction solution, followed by the addition of PVP (2.6 mL, 20 mg mL^−1^ in EG; MW: 55 000). Finally, silver trifluoroacetate (0.8 mL, 282 mM in EG) was added into the mixture using a syringe and under vigorous stirring.

As soon as the silver seeds appeared after the color change to bright yellow, the stirring of the reaction was slowed down and the NPs were allowed to form. Upon reaction completion, the suspension was cooled down by placing the flask into ice. The final nanocubes were purified by repeated centrifugation in acetone (once) and ethanol (three times) and finally redispersed in ethanol.

The as-prepared AgNCs were then used to obtain AuNCs. For this, concentrated nanocubes (the entire batch) were diluted in Milli-Q water (200 mL) after the addition of PVP (1 mg mL^−1^) to prevent their aggregation. An HAuCl_4_ solution (1 mL at a time, 1 mM in Milli-Q water) was gradually added under stirring, and a color change was observed. With the reaction going on, AuNC formation was monitored by recording the UV-vis spectra. The reaction was carried out till the AuNCs did not reach the desirable SPR peak value of around 780–820 nm. Finally, AuNCs were purified by repeated centrifugation and then concentrated. The stock suspension of AuNCs was prepared by dispersing them in Milli-Q water at a particle concentration between 3 and 10 nM depending on the batch.

### AuNCs conjugation with CTX

#### Au-OPEG-CTX preparation

First, CTX was PEGylated using an NHS-PEG_2000_-OPSS heterobifunctional linker. For this, 500 μL CTX (4.344 mg mL^−1^) was diluted in 1 mL Na_2_CO_3_ buffer (10 mM pH 8.5). Then, 148 μL NHS-PEG_2000_-OPSS (0.297 μmol in DMSO) was added at an antibody : PEG ratio of 1 : 20. The reaction was allowed to proceed at 4 °C for 3 h. In the end, the solution was filtered using a Zeba Spin desalting column with 50 kDa cutoff to eliminate the excess linker. The final PEGylated CTX was analyzed by UV-vis spectroscopy and its concentration was calculated using the following parameters: molecular weight of 145.78 kDa and extinction coefficient of 217 440. The final concentration of OPEG-CTX was 1.11 mg mL^−1^. Next, OPEG-CTX was conjugated to AuNCs. For this, 3 mL of 7.7 nM AuNCs (13.99 × 10^12^ NPs) were reacted with 303 μL of 1.114 mg mL^−1^ OPEG-CTX in PBS. The reaction was allowed to proceed overnight at 4 °C. The final nanoconjugates were centrifuged and concentrated. Finally, the conjugated NPs were PEGylated using HS-PEG_2000_-OME. For this, 578 μL PEG (4 mM in MeOH) was added to the NPs (13.99 × 10^12^ NPs), allowed to react overnight, and washed by repeated centrifugation.

#### Au-HC-CTX preparation

First, HC-CTX was prepared by the reduction of cysteine bridges in the antibody. For this, 200 μL CTX (4.344 mg mL^−1^) was reacted with 372 μL cysteamine (1.4 mg mL^−1^) in PBS 1× EDTA buffer. The final reaction volume was 750 μL. The reaction was allowed to proceed at 37 °C for 1.5 h. In the end, the antibody was filtered using a Zeba Spin desalting column with 50 kDa cutoff to eliminate the byproducts. The final HC-CTX was analyzed by UV-vis spectroscopy and its concentration was calculated using the following parameters: molecular weight of 72.89 kDa and the extinction coefficient of 107 720. The final concentration of HC-CTX was 1.023 mg mL^−1^. Next, HC-CTX was conjugated to AuNCs. For this, 3 mL of 7.7 nM AuNCs (13.99 × 10^12^ NPs) was reacted with 165 μL HC-CTX (1.023 mg mL^−1^) in PBS. The reaction was allowed to proceed for 5 h at 4 °C. The final nanoconjugates were centrifuged and concentrated. Finally, the conjugated NPs were PEGylated using HS-PEG_2000_-OME. For this, 578 μL PEG (4 mM in MeOH) was added to the NPs (13.99 × 10^12^ NPs), allowed to react overnight, and washed by repeated centrifugation.

#### Au-spa-CTX preparation

Spa-BC3 protein was conjugated to AuNCs, taking advantage of the three cysteines introduced in the protein sequence. For this, 3 mL of 7.7 nM AuNCs (13.99 × 10^12^ NPs) was reacted with 288 μL of 0.67 mg mL^−1^ in PBS. The reaction was allowed to proceed for 6 h at 4 °C. The final nanoconjugates were centrifuged and concentrated. Then, 77.5 μL of 4.344 mg mL^−1^ of CTX was added and reacted overnight at 4 °C. Au-spa-BC3-CTX nanoconjugates were centrifuged and concentrated. Finally, the conjugated NPs were PEGylated using HS-PEG_2000_-OME. For this, 578 μL PEG (4 mM in MeOH) was added to the NPs (13.99 × 10^12^ NPs), allowed to react overnight, and washed by repeated centrifugation.

#### Au-spa-CTX-linker preparation

First, AuNCs were PEGylated using 75% HS-PEG_2000_-COOH and 25% HS-PEG_3000_-NH_2_. For this, 565 μL HS-PEG_2000_-COOH and 188 μL HS-PEG_3000_-NH_2_ (4 mM in MeOH) were mixed and added to 3 mL of 7.7 nM AuNCs (13.99 × 10^12^ NPs) and allowed to react overnight. The NPs were washed by repeated centrifugation. Next, 8.7 μL (178.5 mg mL^−1^ in DMSO) NHS-PEG-mal linker was added and allowed to react for 2 h at room temperature. The NPs were centrifuged for 30 min at 14 000 rpm. Then, 288 μL of 0.67 mg mL^−1^ spa-BC3 in PBS was added. The reaction was allowed to proceed for 6 h at 4 °C. The final nanoconjugates were centrifuged. Finally, 77.5 μL of 4.344 mg mL^−1^ CTX was added and reacted overnight at 4 °C. The NPs were washed by repeated centrifugation and concentrated.

#### Au-HC-CTX-linker preparation

First, AuNCs were PEGylated using 75% HS-PEG_2000_-COOH and 25% HS-PEG_3000_-NH_2_. For this, 565 μL HS-PEG_2000_-COOH and 188 μL HS-PEG_3000_-NH_2_ (4 mM in MeOH) were mixed and added to 3 mL of 7.7 nM AuNCs (13.99 × 10^12^ NPs) and allowed to react overnight. The NPs were washed by repeated centrifugation. Next, 8.7 μL (178.5 mg mL^−1^ in DMSO) NHS-PEG-mal linker was added and allowed to react for 2 h at room temperature. The NPs were centrifuged for 30 min at 14 000 rpm. Finally, 165 μL HC-CTX (1.023 mg mL^−1^) in PBS was added. The reaction was allowed to proceed for 5 h at 4 °C. The final nanoconjugates were centrifuged and concentrated.

### TEM

TEM micrographs of the AuNCs were obtained on a FEI 120 kV Tecnai G2 Spirit BioTWIN instrument available at the Dipartimento di Scienze Biomediche e Cliniche (University of Milano) at an accelerating voltage of 120 kV. The samples were prepared by evaporating a drop of NPs onto a carbon-coated copper grid and allowing it to dry in air. The average particle diameter was obtained by measuring about 150–200 particles by using the Image-J software.

### Cellular uptake by TEM

MDA-MB-231 and MDA-MB-468 cells were cultured on a 6-well dish at a density of 1 × 10^6^ cells per well. The cells were incubated with Au-spa-CTX and Au-OPEG-CTX NCs for 24 h. After incubation, the medium containing excess NPs was removed and the cells were washed several times with PBS, detached, and centrifuged at 2000 rpm for 5 min. The primary fixation of the cells was carried out with a 0.1 M PBS solution of 2.5% glutaraldehyde. Then, the cells were washed with PBS and post-fixed using a 1.5% aqueous solution of OsO_4_ (**caution**: extremely toxic) for 2 h followed by successively washing with PBS. Then, the cells were dehydrated using increasing concentrations of ethanol (30–100%). Finally, the cell pellets were infiltrated with a mixture of 1 : 1 epoxy resin in 100% ethanol overnight and then left to polymerize at 60 °C for 48 h. Ultrathin sections (∼70 nm) were cut using a diamond knife. The sections were stained with uranyl acetate and lead citrate solutions and imaged using the FEI 120 kV Tecnai G2 Spirit BioTWIN instrument on 120 kV.

### SDS–PAGE analysis

The SDS–PAGE analysis was performed using a Mighty Small II SE 250 Hoefer Scientific Instruments gel electrophoresis apparatus (San Francisco, CA). For the analysis, 3 different amounts of spa-BC3-CTX complex (1, 3, and 5 μg) were loaded onto the gel, previously quantified by the Bradford colorimetric assay. Here, 1 μg of free spa-BC3 and CTX were used as the positive controls. The samples, including the controls, were treated with 0.2 volumes (5 μL) of the sample buffer, heated to 100 °C for 10 min, and then loaded onto the gel. The gel was immersed in the running buffer (1×) containing 10% SDS. The electrophoretic run was conducted at constant amperage of 25 mA. The gel was washed under constant stirring with deionized water and subsequently stained by the Imperial Protein Stain® (Thermo Scientific) following the manufacturer's protocol. Finally, the colored gel was washed again with deionized water and visualized.

### DLS

DLS measurements were performed using a Zetasizer Nano Instrument from Malvern Instruments Ltd. (Amesbury, UK) operating at 4 mW with a He–Ne laser (633 nm) using a scattering angle of 90°. A disposable cuvette (optical path length: 1 cm) was used for the measurements. The cuvettes were cleaned with Milli-Q water and stored to dry. The samples were prepared by dilution with Milli-Q water or 10 mM NaCl, followed by filtration with a 0.2 μm cellulose acetate syringe filter before loading into the cuvette in order to remove large interfering particulate matter. Each sample was allowed to equilibrate for 1 min prior to starting the measurements. Three to ten independent measurements of 60 s duration were performed at 25 °C. The calculations of hydrodynamic diameter were performed using Mie theory, where the absolute viscosity and refractive index values of the medium were considered to be 0.911 cP and 1.334, respectively. The refractive index of the material was set to 0.2, Abs 3.32.

### Zeta potential measurements

The zeta potential was determined at 25 °C using a Zetasizer Nano Instrument from Malvern Instruments Ltd. (Amesbury, UK). The samples for measurements were prepared by concentrated NPs in 10 mM NaCl. A minimum of 3 runs and 10 subruns per sample were performed to establish measurement repeatability. The zeta potential was automatically calculated from the electrophoretic mobility based on the Smoluchowski theory. A viscosity of 0.891 cP, dielectric constant of 78.6, and Henry function of 1.5 were used for the calculations.

### NTA

NP concentration was measured by NTA using a NanoSight Model NS300 (Malvern Instruments Ltd., Amesbury, UK) equipped with a 532 nm laser excitation source and high-sensitivity scientific CMOS camera. NTA 3.0 was used for data collection and analysis. The samples were prepared by diluting the original NPs in ultrapure sterile water. The samples were manually injected into the sample chamber using 1 mL silicon oil-free plastic syringes. All the video capture and analysis settings, including camera shutter, camera gain, and detection threshold, remained identical for all the samples in an individual experiment. All the samples that were collected and analyzed had more than 200 completed tracks. A minimum of 5 independent measurements were performed per sample. Final NP concentration was calculated and expressed in nM.

### UV-vis spectroscopy

UV-vis spectra of NP solutions in the 200–840 nm range were recorded on a Nanodrop 2000c spectrometer using quartz cuvettes (path length: 1 cm). The solutions for UV-vis spectroscopy analysis were obtained by diluting the original NPs to the desired concentration with Milli-Q water. AuNC stability was studied by UV-vis spectroscopy by incubating NPs in 100% FBS, 150 mM NaCl, carbonate (pH 8.5), and PBS (pH 7.4) buffers. For this, an aliquot of concentrated NPs was dispersed in an appropriate medium and incubated for 20 min at room temperature. Subsequently, the absorption spectra of NPs in the 500–900 nm range were measured.

### Cell culture

The cells were provided by LGC Standards, Italy. The culture medium for MDA-MB-231 human breast cancer cells was MEM with Earle's salts, supplemented with 10% FBS, l-glutamine (2 mM), penicillin (50 mg mL^−1^), and streptomycin (50 mg mL^−1^). The culture medium for MDA-MB-468 human breast cancer cells was DMEM/Ham's F-12, supplemented with 10% FBS, l-glutamine (2 mM), penicillin (50 mg mL^−1^), and streptomycin (50 mg mL^−1^). The culture medium for 3T3-L1 murine fibroblast cells was DMEM (high glucose), supplemented with 10% FBS, l-glutamine (2 mM), penicillin (50 mg mL^−1^), and streptomycin (50 mg mL^−1^). The cells were grown at 37 °C in a humidified CO_2_ incubator in a monolayer. The confluent cells were harvested with 0.05% trypsin/0.02% EDTA and were seeded in Petri dishes for all the experiments, which were performed using sub-confluent cell cultures.

### Cellular uptake by ICP-AES

The cells were cultured on a 6-well dish at a density of 7 × 10^5^ cells per well. The cells were incubated with the nanoconjugates for 24 h. After incubation, the medium containing excess NPs was removed and the cells were washed several times with PBS, detached, and centrifuged at 2000 rpm for 5 min. Thereafter, the cells were counted by a hemocytometer and digested in 4 mL freshly prepared aqua regia (**caution**: extremely corrosive). Two days later, the samples were diluted with Milli-Q water to a final volume of 15 mL. The gold content was determined by ICP-AES. A sample of AuNCs at a known concentration (NPs per mL) used for incubation was analyzed by ICP-AES in addition to calculating the number of gold atoms per particle. The results were expressed as the number of gold NPs per cell, considering a uniform distribution of NPs.

### Binding studies by ICP-AES

For the binding studies, the cells were cultured on a 6-well dish at a density of 7 × 10^5^ cells per well. Prior to incubation with AuNCs, the cells were preincubated with CTX (1 mg mL^−1^ in 1% PBS-BSA) for 30 min at 4 and 37 °C. Free CTX was removed by washing the cells in PBS, and the cells were incubated with Au-spa-CTX and Au-OPEG-CTX NCs for 1 h at 4 and 37 °C. After multiple washings from NP excess, the cells were processed for ICP-AES analysis as described.

## Conflicts of interest

There are no conflicts to declare.

## Supplementary Material

NA-001-C9NA00241C-s001

## References

[cit1] Zugazagoitia J., Guedes C., Ponce S., Ferrer I., Molina-Pinelo S., Paz-Ares L. (2016). Clin. Ther..

[cit2] Lacombe S., Porcel E., Scifoni E. (2017). Cancer Nanotechnol..

[cit3] Dilnawaz F., Acharya S., Sahoo S. K. (2018). Int. J. Pharm..

[cit4] Overchuk M., Zheng G. (2018). Biomaterials.

[cit5] Colombo M., Fiandra L., Alessio G., Mazzucchelli S., Nebuloni M., De Palma C., Kantner K., Pelaz B., Rotem R., Corsi F., Parak W. J., Prosperi D. (2016). Nat. Commun..

[cit6] Sivaram A. J., Wardiana A., Howard C. B., Mahler S. M., Thurecht K. J. (2018). Adv. Healthcare Mater..

[cit7] Skrabalak S. E., Chen J., Sun Y., Lu X., Au L., Cobley C. M., Xia Y. (2008). Acc. Chem. Res..

[cit8] Avvakumova S., Galbiati E., Sironi L., Locarno S. A., Gambini L., Macchi C., Pandolfi L., Ruscica M., Magni P., Collini M., Colombo M., Corsi F., Chirico G., Romeo S., Prosperi D. (2016). Bioconjugate Chem..

[cit9] Klein S., Petersen S., Taylor U., Rath D., Barcikowski S. (2010). J. Biomed. Opt..

[cit10] Liu T.-C., Jin X., Wang Y., Wang K. (2017). Am. J. Cancer Res..

[cit11] Liu K. C., Arivajiagane A., Wu S. J., Tzou S. C., Chen C. Y., Wang Y. M. (2019). Nanomedicine.

[cit12] Ashton J. R., Gottlin E. B., Patz E. F., West J. L., Badea C. T. (2018). PLoS One.

[cit13] Qiu M., Xu L., Tian Y., Zhang Y., Wu Q., Gu N., Li S., Yin R., Qian Y. (2014). Sci. Rep..

[cit14] Sreeranganathan M., Uthaman S., Sarmento B., Mohan C. G., Park I.-K., Jayakumar R. (2017). Int. J. Nanomed..

[cit15] Li W., Ji Y. H., Li C. X., Liu Z. Y., Li N., Fang L., Chang J., Tan J. (2016). World J. Gastroenterol..

[cit16] Löw K., Wacker M., Wagner S., Langer K., Von Briesen H. (2011). Nanomedicine.

[cit17] Cho Y. S., Yoon T. J., Jang E. S., Soo Hong K., Young Lee S., Ran Kim O., Park C., Kim Y. J., Yi G. C., Chang K. (2010). Cancer Lett..

[cit18] Li C., Tan J., Chang J., Li W., Liu Z., Li N., Ji Y. (2017). Oncol. Rep..

[cit19] Gelfand A. A., Fullerton H. J., Jacobson A., Sidney S., Goadsby P. J., Kurth T., Pressman A. (2015). Cephalalgia.

[cit20] Kaluzova M., Bouras A., Machaidze R., Hadjipanayis C. G. (2015). Oncotarget.

[cit21] Zhang X., Li Y., Wei M., Liu C., Yang J. (2019). Drug Delivery.

[cit22] Catrambone F., Colombo M., Corsi F., Truffi M., Prosperi D., Giustra M., Rizzuto M. A., Garbujo S., Pacini C., Pandolfi L., Bonizzi A., Fiandra L., Monieri M., Mazzucchelli S. (2018). Bioconjugate Chem..

[cit23] Roncato F., Rruga F., Porcù E., Casarin E., Ronca R., Maccarinelli F., Realdon N., Basso G., Alon R., Viola G., Morpurgo M. (2018). Nat. Commun..

[cit24] Sousa F., Castro P., Fonte P., Kennedy P. J., Neves-Petersen M. T., Sarmento B. (2017). Expert Opin. Drug Delivery.

[cit25] Montenegro J. M., Grazu V., Sukhanova A., Agarwal S., de la Fuente J. M., Nabiev I., Greiner A., Parak W. J. (2013). Adv. Drug Delivery Rev..

[cit26] Algar W. R., Prasuhn D. E., Stewart M. H., Jennings T. L., Blanco-Canosa J. B., Dawson P. E., Medintz I. L. (2011). Bioconjugate Chem..

[cit27] Avvakumova S., Colombo M., Tortora P., Prosperi D. (2014). Trends Biotechnol..

[cit28] García-Fernández L., Garcia-Pardo J., Tort O., Prior I., Brust M., Casals E., Lorenzo J., Puntes V. F. (2017). Nanoscale.

[cit29] Colombo M., Mazzucchelli S., Montenegro J. M., Galbiati E., Corsi F., Parak W. J., Prosperi D. (2012). Small.

[cit30] Mazzucchelli S., Colombo M., Verderio P., Rozek E., Andreata F., Galbiati E., Tortora P., Corsi F., Prosperi D. (2013). Angew. Chem., Int. Ed..

[cit31] Colombo M., Sommaruga S., Mazzucchelli S., Polito L., Verderio P., Galeffi P., Corsi F., Tortora P., Prosperi D. (2012). Angew. Chem., Int. Ed..

[cit32] Chen H. L., Hsu F. T., Kao Y. C. J., Liu H. S., Huang W. Z., Lu C. F., Tsai P. H., Ali A. A. A., Lee G. A., Chen R. J., Chen C. Y. (2017). J. Nanobiotechnol..

[cit33] Polito L., Monti D., Caneva E., Delnevo E., Russo G., Prosperi D. (2008). Chem. Commun..

[cit34] Occhipinti E., Verderio P., Natalello A., Galbiati E., Colombo M., Mazzucchelli S., Salvadè A., Tortora P., Doglia S. M., Prosperi D. (2011). Nanoscale.

[cit35] Bartczak D., Kanaras A. G. (2011). Langmuir.

[cit36] Kocbek P., Obermajer N., Cegnar M., Kos J., Kristl J. (2007). J. Controlled Release.

[cit37] Zhang Q., Li W., Wen L. P., Chen J., Xia Y. (2010). Chem.–Eur. J..

[cit38] Pal S. K., Childs B. H., Pegram M. (2011). Breast Cancer Res. Treat..

[cit39] Avvakumova S., Galbiati E., Pandolfi L., Mazzucchelli S., Cassani M., Gori A., Longhi R., Prosperi D. (2014). Bioconjugate Chem..

[cit40] Krpetić Z., Saleemi S., Prior I. A., Sée V., Qureshi R., Brust M. (2011). ACS Nano.

[cit41] Lowery A. R., Gobin A. M., Day E. S., Halas N. J., West J. L. (2006). Int. J. Nanomed..

[cit42] Chattopadhyay N., Cai Z., Pignol J.-P., Keller B., Lechtman E., Bendayan R., Reilly R. M. (2010). Mol. Pharm..

[cit43] Bergeron E., Boutopoulos C., Martel R., Torres A., Rodriguez C., Niskanen J., Lebrun J.-J., Winnik F. M., Sapieha P., Meunier M. (2015). Nanoscale.

[cit44] Tan Y. H., Liu M., Nolting B., Go J. G., Gervay-hague J., Liu G. (2008). ACS Nano.

[cit45] Bachelet M., Chen R. (2016). Chem. Commun..

[cit46] Wu S., Liu H., Liang X. M., Wu X., Wang B., Zhang Q. (2014). Anal. Chem..

[cit47] Fiandra L., Mazzucchelli S., De Palma C., Colombo M., Allevi R., Sommaruga S., Clementi E., Bellini M., Prosperi D., Corsi F. (2013). ACS Nano.

[cit48] Cova E., Colombo M., Inghilleri S., Morosini M., Miserere S., Penarada-Avila J., Santini B., Piloni D., Magni S., Gramatica F., Prosperi D., Meloni F. (2015). Nanomedicine.

[cit49] Mazzucchelli S., Colombo M., De Palma C., Salvadè A., Verderio P., Coghi M. D., Clementi E., Tortora P., Corsi F., Prosperi D. (2010). ACS Nano.

[cit50] Colombo M., Mazzucchelli S., Collico V., Avvakumova S., Pandolfi L., Corsi F., Porta F., Prosperi D. (2012). Angew. Chem., Int. Ed..

[cit51] Jansson B., Uhlén M., Nygren P. Å. (1998). FEMS Immunol. Med. Microbiol..

[cit52] Jendeberg L., Nilsson P., Larsson A., Denker P., Uhlén M., Nilsson B., Nygren P. Å. (1997). J. Immunol. Methods.

[cit53] Valdepérez D., del Pino P., Sánchez L., Parak W. J., Pelaz B. (2016). J. Colloid Interface Sci..

[cit54] Sokolov K., Follen M., Aaron J., Pavlova I., Malpica A., Lotan R., Richards-kortum R. (2003). Cancer Res..

[cit55] Kim T., Lee C. H., Joo S. W., Lee K. (2008). J. Colloid Interface Sci..

[cit56] Zhang M., Kim H. S., Jin T., Woo J., Piao Y. J., Moon W. K. (2017). Oncotarget.

[cit57] Jeong H., Kim J., Lee Y., Seo J. H., Hong S. R., Kim A. (2014). Oncol. Rep..

[cit58] Rabanel J. M., Hildgen P., Banquy X. (2014). J. Controlled Release.

[cit59] Pereira Gomes C., Leiro V., Ferreira Lopes C. D., Spencer A. P., Pêgo A. P. (2018). Acta Biomater..

[cit60] Siahpoush V., Ahmadi-kandjani S., Nikniazi A. (2018). Opt. Commun..

[cit61] Skrabalak S. E., Au L., Li X., Xia Y. (2007). Nat. Protoc..

