# Supplementary material for: Does conjugation strategy matter? Cetuximab-conjugated gold nanocages for targeting triple-negative breast cancer cells
Source: Nanoscale Adv. 2019 Jul 23;1(9):3626–38. doi: 10.1039/c9na00241c (PMC9419579; doi:10.1039/c9na00241c)
Supplement: NA-001-C9NA00241C-s001 [file NA-001-C9NA00241C-s001.pdf]

Electronic Supplementary Material (ESI) for Nanoscale Advances.
This journal is © The Royal Society of Chemistry 2019

Electronic Supplementary Information

Does conjugation strategy matter? Cetuximab conjugated gold nanocages for triple negative breast cancer cells targeting

S. Avvakumova,[a] L. Pandolfi,[b] E. Soprano,[a] L. Moretto,[a] M. Bellini,[a] E. Galbiati,[a] M. A. Rizzuto,[a] M. Colombo,[a] R. Allevi,[c] F. Corsi,[c,d,e] A. Sánchez Iglesias,[f] and D. Prosperi*[a]

[a] *University of Milano "Bicocca", Department of Biotechnology and Bioscience, Piazza della Scienza, 2, 20126, Milano, Italy*
[b] *Clinica di Malattie dell'Apparato Respiratorio, IRCCS Fondazione Policlinico San Matteo, Pavia, Italy*
[c] *Dipartimento di Scienze Biomediche e Cliniche "Luigi Sacco", Università di Milano, via G.B. Grassi 74, 20157 Milano, Italy*
[d] *Surgery Department, Breast Unit, ICS Maugeri S.p.A. SB, via S. Maugeri 10, Pavia, Italy*
[e] *Nanomedicine Laboratory, ICS Maugeri S.p.A. SB, via S. Maugeri 10, Pavia, Italy*
[f] *CIC BiomaGUNE and CIBER-BBN, Paseo de Miramón 182, 20014 Donostia-San Sebastián, Spain*

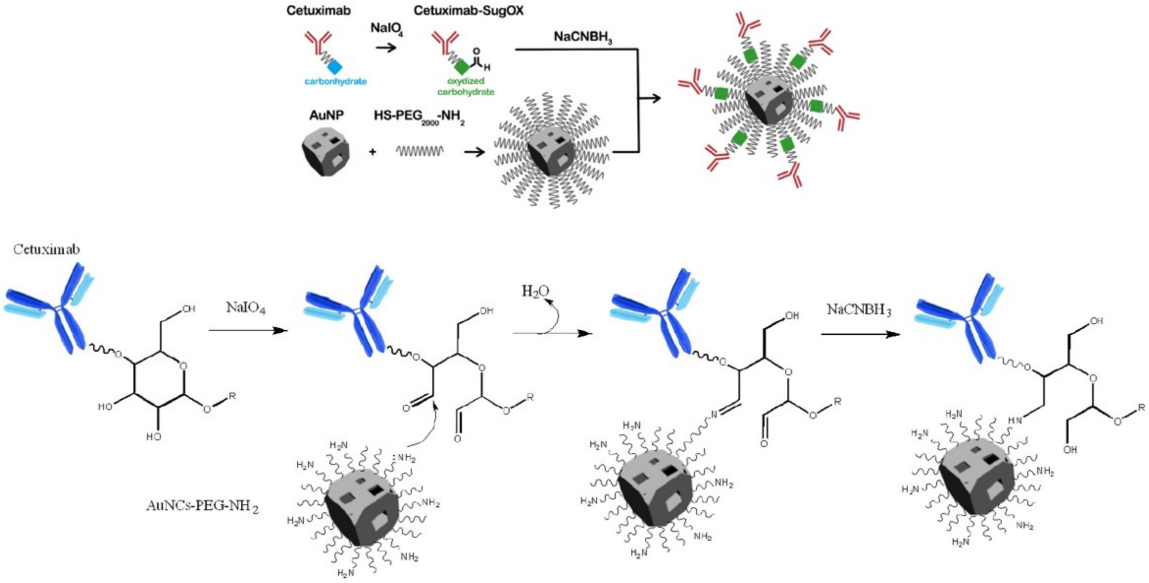

Figure S1. Au-SugOX NCs preparation *via* sugar moiety oxidation of CTX.

Table S1. SPR band maxima of AuNPs before functionalization (AuNCs[1] and AuNCs[2]) and after functionalization with CTX. SPR peaks shift after functionalization. Numbers [1] and [2] represent two different batches of staring nanoparticles, showing that SPR shift is independent of the batch used.

Sample	SPR peak, nm	δ (SPR), nm
AuNCs[1]	732 nm	--
AuNCs[2]	756 nm	--
OPEG CTX[2]	772 nm	16 nm
spa-CTX[1]	744.5 nm	12.5 nm
spa-CTX LINKER[1]	748 nm	16 nm
HC CTX[1]	749 nm	17 nm
HC CTX LINKER[1]	748 nm	16 nm

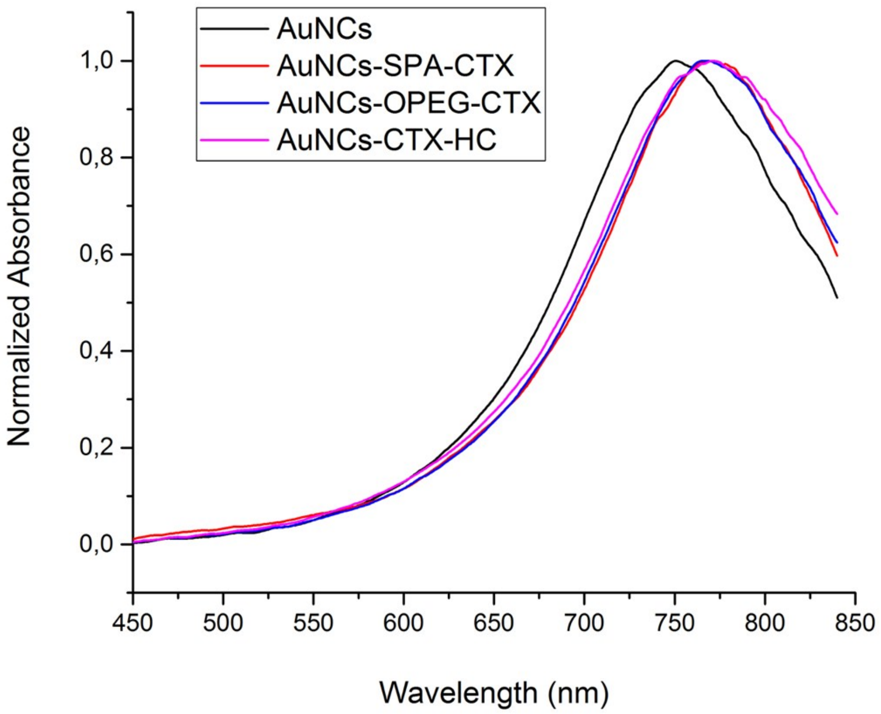

Figure S2. UV-vis spectra of starting AuNCs, Au-OPEG-CTX, Au-spa-CTX and Au-HC-CTX after conjugation with CTX.

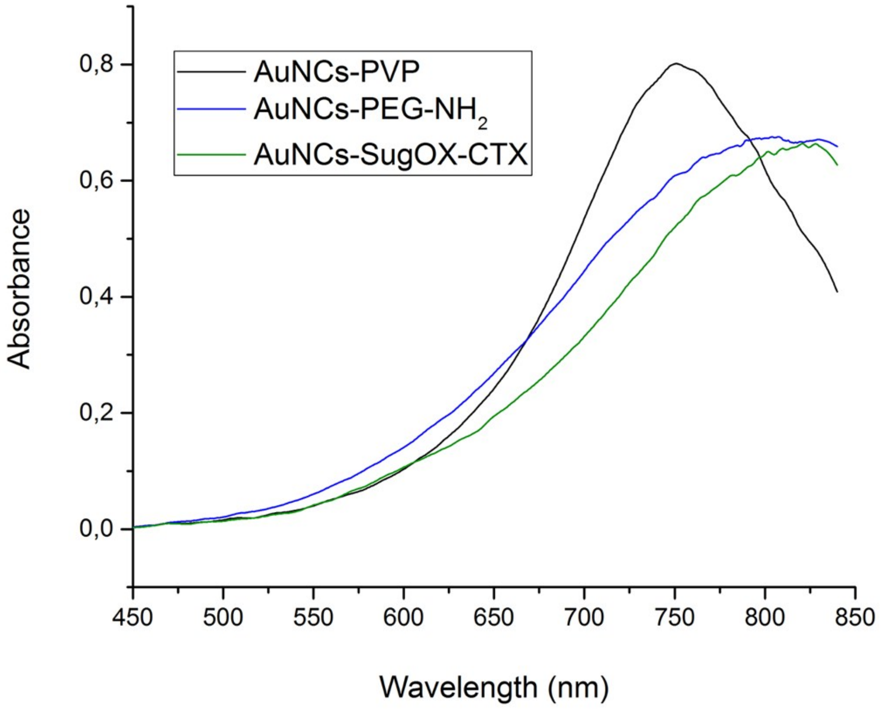

Figure S3. UV-vis spectra of starting AuNCs, AuNCs PEGylated with HS-PEG_{3000}-NH_2 (Au-PEG-NH_2), and functionalized with CTX with oxidized saccharide moiety (Au-SugOX-CTX).

Table S2. Dynamic light scattering and zeta potential values of CTX conjugated AuNCs.

**Sample**	**Number mean, nm**	**PdI**	**Z-Pot, mV**
Au-PVP	53.87±3.77	0.144±0.011	-26.0±2.7
Au-OPEG-CTX	63.36±1.89	0.170±0.006	-19.7±0.8
Au-spa-CTX	55.52±3.61	0.154±0.008	-19.3±1.6
Au-linker-spa-CTX	68.03± 3.423	0.164± 0.016	-25.9± 0.231
Au-HC-CTX	84.65±8.69	0.173±0.014	-16.7±0.3
Au-linker-HC-CTX	59.79± 5.28	0.219± 0.009	-22.8± 0.141

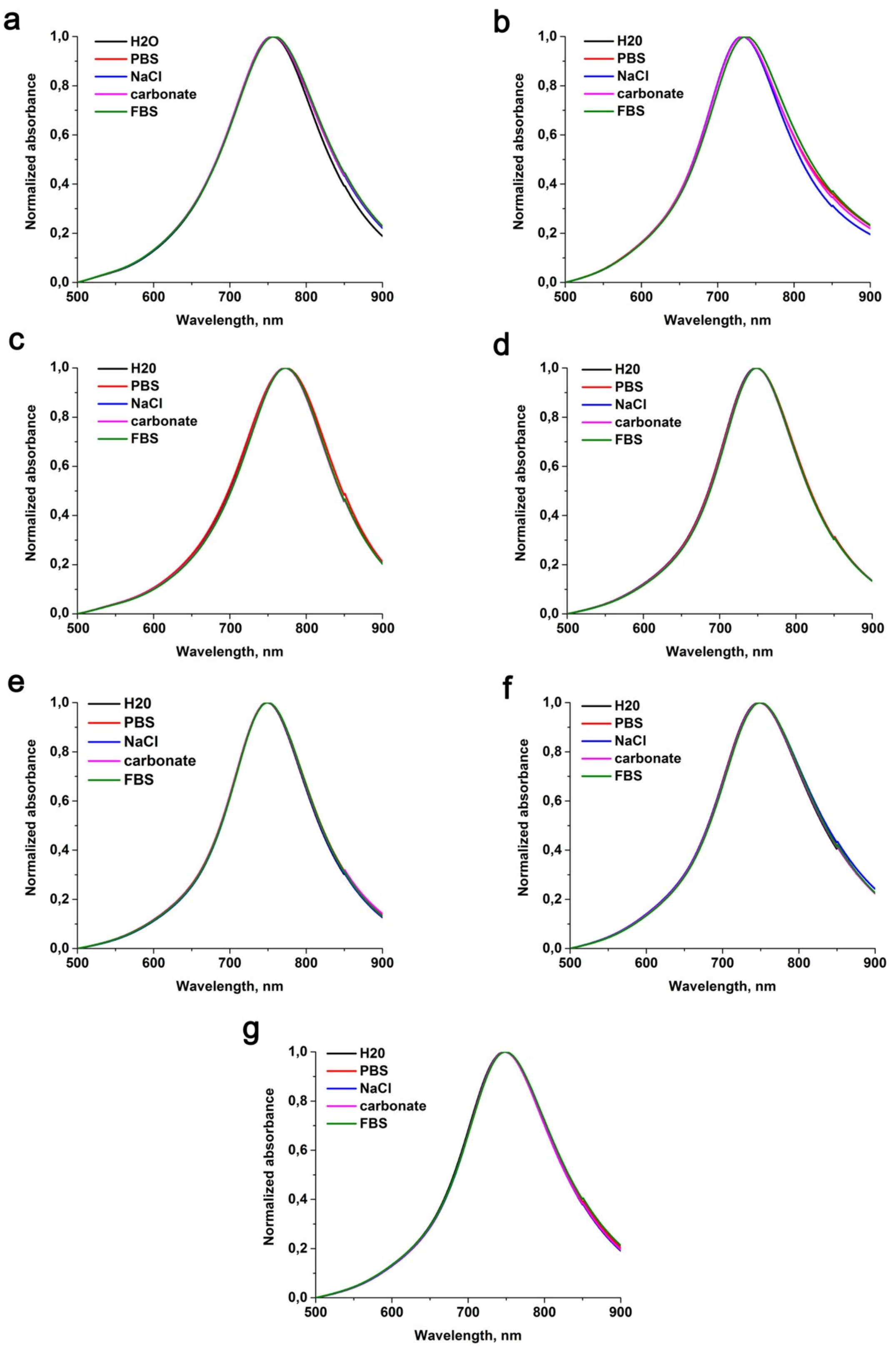

Figure S4. Colloidal stability of nanoparticles in PBS pH 7.5, NaCl 150 mM, carbonate buffer pH 8.5, and FBS: a) Au-PVP (756 nm SPR), b) Au-PVP (732 nm SPR), c) Au-OPEG-CTX, d) Au-spa-CTX, e) Au-HC-CTX, f) Au-spa-linker-CTX, and g) Au-HC-linker-CTX.

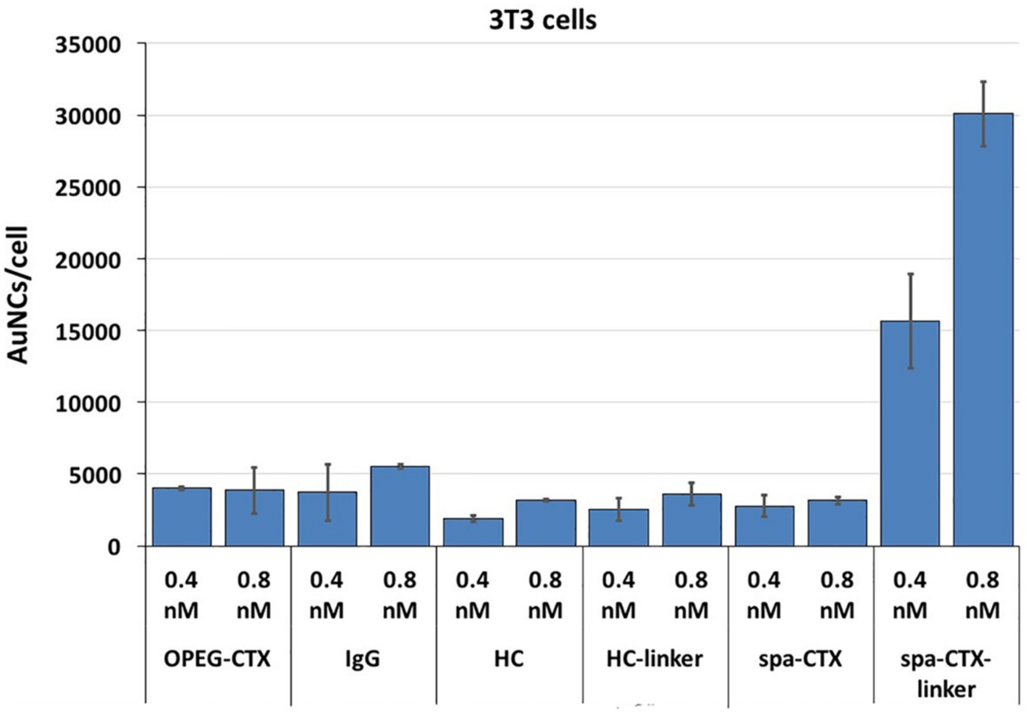

Figure S5. Cellular uptake of AuNCs conjugated with CTX by different conjugation approaches in 3T3-L1 cells. Gold content per cell was analysed by ICP-AES, and number of NPs per cell was calculated. 0.4 and 0.8 nM concentration of nanoconjugates was used for the experiments.

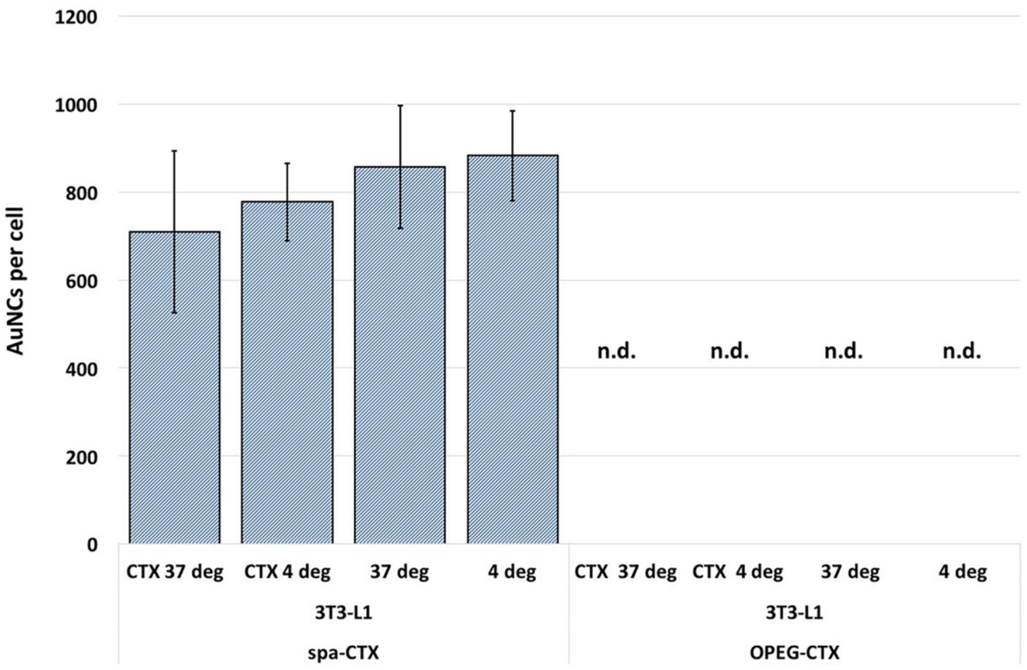

Figure S6. Cellular binding and uptake of Au-OPEG-CTX and Au-spa-CTX nanoconjugates at 4 and 37 °C in 3T3-L1 cells, in presence and absence of free CTX.
